# Changing the Drug Delivery System: Does it Add to Non-Compliance Ramifications Control? A Simulation Study on the Pharmacokinetics and Pharmacodynamics of Atypical Antipsychotic Drug

**DOI:** 10.3390/pharmaceutics12040297

**Published:** 2020-03-25

**Authors:** Mohammed H. Elkomy

**Affiliations:** 1 Department of Pharmaceutics, College of Pharmacy, Jouf University, Sakaka 42421, Saudi Arabia; mhalkomy@ju.edu.sa; Tel.: +966-560967705; 2 Department of Pharmaceutics and Industrial Pharmacy, Beni-Suef University, Beni-Suef 62511, Egypt

**Keywords:** antipsychotic, schizophrenia, pharmacokinetic/pharmacodynamic modeling, compliance, Monte Carlo simulations, extended-release

## Abstract

This study investigates the pharmacokinetic (PK) and pharmacodynamic (PD) consequences of shifting from Quetiapine fumarate immediate-release (IR) to extended-release (XR) formulation in non-adherent schizophrenia patients. Monte-Carlo simulations using population PK and PD models were implemented to predict the time course of plasma concentration and Brief Psychiatric Rating Scale (BPRS) scores following the oral administration of 200 mg Seroquel^®^ every 12 h and 400 mg Seroquel XR^®^ every 24 h in patients experiencing dose delay, omission or doubling. Parameters were computed and their distributions were compared using the Kolmogorov–Smirnov test. Dose irregularities with both formulations had different effects on plasma concentration and %reduction in BPRS scores from baseline. However, the odds ratio of getting a %reduction in BPRS below 14%, or plasma concentration exceeding 500 µg/L, were comparable for adherent and non-adherent patients. Plasma therapeutic concentration after treatment cessation was maintained for <24 h in 48% and 29.6% of patients, and a steady state recovery time of <48 h was achieved in 51% and 13.4% of patients on the IR and XR formulations, respectively. Monte-Carlo simulations predict that the risks associated with the IR dose irregularities are not worsened when the XR formulation is used instead. Non-adherence events involving a single dose of either formulation do not require rescue doses.

## 1. Introduction

Dose compliance (also known as dose adherence) means the strict commitment of the patient to the medication dosage regimen as prescribed. Therefore, dose delay or omission, therapy discontinuation, or even shifting to a different regimen at the patient’s discretion is considered dose noncompliance or non-adherence. In diseases requiring long-term or life-long treatment, dose adherence is a serious issue. Non-adherence can lead to drug plasma concentration undershoot (i.e., trough below minimum effective concentration) or overshoot (i.e., peak above maximum tolerated concentration). This undesirable behavior is particularly problematic in morbid diseases such as schizophrenia, where departure from the prescribed doses has been associated to symptom exacerbation, psychotic state relapse, diminished cognitive capacity, violence, hospitalization, and, most importantly, increased risk of suicide [1,2,3,4]. It is quite alarming that non-adhesion to antipsychotic drugs (ASD) was cited as the most common cause for admittance to psychiatric hospitals [5].

More than 50% of patients with schizophrenia fail to adhere to the prescribed dosage regimens of ASDs [1,2,6]. This phenomenon has been related to ASD inefficiency, intolerability and costs [1,2]. Unintentional non-adherence due to forgetfulness was identified as the major reason for non-adherence in a group of schizophrenic patients [7] and patients with bipolar disorder [8], accounting for 36.2% and 55% of non-adherent patients in both cases, respectively.

Schizophrenic patients experiencing unintentional non-adherence may benefit from shifting from complex dosage regimens that involve three times (TID) or two times (BID) daily dosed, immediate-release (IR) formulations to the corresponding once-daily (QD) dosed, extended release (XR) formulations. However, missing a dose from a QD-dosed drug is equivalent to dropping 100% of the daily dose versus 50% and 33.3% in the case of BID- and TID-dosed drugs, respectively. Theoretically, dosing irregularities constituting 100% of the daily dose are expected to produce more prominent perturbations in the drug’s plasma concentrations, and hence will result in more aggravated health consequences [9]. This perspective is antagonized by another, that sees XR formulations as a way to blunt the influence of dosing irregularities on the drug’s plasma concentration [10]. Theoretically, XR formulations have the potential to preserve plasma therapeutic levels for prolonged time intervals owing to slower absorption rates.

Quetiapine is atypical ASD that has been approved for treatment of schizophrenia and management of the manic and depressive episodes of bipolar disorder [11]. Quetiapine fumarate is available in multiple formulations including an IR formulation (Seroquel^®^) and XR formulation (Seroquel XR^®^) [12]. The pharmacokinetic profiles of the BID Seroquel^®^ and the QD Seroquel XR^®^ formulations were bioequivalent based on area under curve comparison, although steady-state peak plasma concentration was approximately 13% lower for the XR formulation [13].

To evaluate the consequences of shifting from Seroquel^®^ to Seroquel XR^®^ on the pharmacokinetic (PK) and pharmacodynamic (PD) properties of Quetiapine in non-adherent patients, clinical investigation is warranted. However, it is neither ethical nor practical to ask patients with schizophrenia to intentionally delay, omit or double their prescribed doses for the sake of a clinical study. Additionally, it is not practical to conduct a clinical trial that involves administering multiple doses of Quetiapine to healthy volunteers to achieve steady-state. Accordingly, in silico approaches based on PK/PD modeling and simulations are the feasible path to evaluate dose irregularities [10,14].

Since the late 1990s, PK/PD modeling and simulations have been a powerful tool to recommend dose regimens in vulnerable populations [15,16,17,18,19,20]. However, few studies have used this tool to assess the risks associated with dose noncompliance in different treatments [21], dosage regimens [14,22], and formulations [10,23], and to recommend replacement (rescue) doses to minimize these risks [14]. This study aims to compare the PK and PD responses to delayed, omitted, and doubled dosing with the Quetiapine IR and XR formulations. The ultimate goal was to explore through simulations if replacing IR formulations with equivalent XR formulations worsens the consequences of Quetiapine dose irregularities.

## 2. Methods

### 2.1. Population Pharmacokinetic (PK) Model

A previously reported population PK model for Seroquel^®^ and Seroquel XR^®^ [12] was used to simulate the time course of Quetiapine plasma concentrations. The model was developed using steady-state data from six Phase I studies contributing 4367 concentrations from 87 western and 40 Chinese patients diagnosed with schizophrenic disorders. The patients were 18–65 (average 39.8) years old weighing 45–124.5 (average 80) kg. Seroquel^®^ dose regimens were 150, 200, 300, and 400 mg BID. Seroquel XR^®^ dose regimens were 50, 200, 300, 400, 600, and 800 mg QD. The collected blood samples were assayed using a validated LC-MS method.

The non-linear mixed effects software (NONMEM version 7.2, ICON, Ellicott City, MD, USA) was used to build the population model using the First-order Conditional Estimation with an interaction of intra- and inter-individual variability (FOCE-I). The structural model was a 1-compartment model with first-order absorption and first-order elimination. An exponential model was used for the inter-individual variability. A mixed additive and proportional error model was used for the residual variability. The model was internally validated using the visual predictive check and bootstrap analyses. The model was externally validated by predicting the Quetiapine PK profiles reported in the literature.

### 2.2. Population Pharmacodynamics (PD) Model

A previously published population PD model was used to simulate the time course of Brief Psychiatric Rating Scale (BPRS) under the influence of placebo administration and treatment with Seroquel^®^ [24]. The model was developed using PK/PD data from a Phase II trial with 295 patients contributing 1913 BPRS records. The patients were allocated to seven treatment arms assigned to escalating doses of between 75 and 750 mg per day. The PD response was assessed every week for 6 weeks.

The population model was built using NONMEM version 1.0 (University of California, San Francisco, CA, USA). A linear model in time best described the placebo effect and an *E_max_* model best described the inhibitory effect of the drug. Normal and log-normal distributions were used for the inter-individual variability. A proportional error model was used for the residual variability. The model was validated internally by the posterior predictive check analysis and externally by predicting the outcomes of a Phase III clinical trial.

### 2.3. Simulation Scenarios

Simulation scenarios were based on a 42-year-old schizophrenic patient (the typical age used in the population PK model [12]). The patient received a 400 mg total daily dose of Seroquel^®^ (as 200 mg every 12 h) or Seroquel XR^®^ (as 400 mg every 24 h). The simulated dosage regimens were assumed to provide full control over schizophrenia symptoms without adverse events. Additionally, the simulations assumed that steady state plasma level was attained. The simulated scenarios were as follows:

A. Dose adherence: The patient administers 200 mg of the IR formulation every 12 h (200 mg BID) and 400 mg of the XR formulation every 24 h (400 mg QD) on a regular basis without mistakes or cessation;
B. Dose delay: One dose is administered at 25%, 50%, 75%, and 100% of the dosing interval (equivalent to 3, 6, 9, and 12 h for the BID and 6, 12, 18, and 24 h for the QD), with the next dose to be administered as scheduled;
C. Dose omission: One dose is missed without replacement;
D. Dose doubling: One dose is replicated twice by mistake;
E. Dose discontinuation: The patient stops drug administration.

### 2.4. Monte-Carlo Simulations

Monte-Carlo (MC) simulations with the population PK and PD models were conducted in *R* version 3.5.2 (*R* Foundation for Statistical Computing, Vienna, Austria). The fixed-effects and one-level of the nested random effects (the between subject variability) were used in the simulation process. The codes implemented in the simulations are provided in the Supplementary Material. The programmed equations for the PK model were driven from Zhou et al. [12] and were as follows

(1) $$C_{p}=\frac{D_{0}\times k_{a}}{V\times \left(k_{a}-k_{e}\right)}\left(e^{-k_{e}\times time}-e^{-k_{a}\times time}\right)$$

where *C_p_* is the plasma concentration in µg/L; *D_0_* is the administered dose in µg; *V* is the distribution volume in L; *k_a_* is the absorption rate constant in h^−1^; and *k_e_* is the elimination rate constant in h^−1^. The parameters were sampled from the following log-normal distribution, assuming independence,

(2) lnθ~N(lnμ,σ)

where θ is the PK parameters, µ is the estimated fixed-effects, and σ is the standard deviation of between-subject random effects.

(3) $$\theta =\left[\begin{array}{c} k_{e}\\ \begin{array}{c} k_{a}\left(IR\right)\\ \begin{array}{c} k_{a}\left(XR\right)\\ V \end{array} \end{array} \end{array}\right]\;,\;\mu =\left[\begin{array}{c} 0.12\\ \begin{array}{c} 1.46\\ \begin{array}{c} 0.1\\ 573.7 \end{array} \end{array} \end{array}\right]\;,\;\sigma =\left[\begin{array}{c} 0.40\\ \begin{array}{c} 0.75\\ \begin{array}{c} 1.50\\ 0.47 \end{array} \end{array} \end{array}\right]$$

The programmed equations for the PD model were driven from Kimko et al. [24] and were as follows

(4) $$BPRS=BPRS_{0}+\alpha \times time-\frac{E_{max}\times C_{p}}{EC_{50}+C_{p}}$$

where *BPRS_0_* is the baseline BPRS score, *α* is the slope of the placebo effect in h^−1^, *E_max_* is the maximum change in BPRS scores from the baseline due to treatment, and *EC_50_* is the concentration associated with 50% of the maximum drug effect in µg/L. The parameters were sampled from the following distributions

(5) $$lnBPRS_{0}\sim N\left(3.65,0.27\right)$$

(6) α~N(−0.008,0.0001)

(7) $$lnE_{max}\sim N\left(2.2,0.41\right)$$

(8) $$lnEC_{50}\sim N\left(4.42,1.77\right)$$

One thousand virtual patients with Quetiapine time–plasma concentration–BPRS profiles following the administration of the IR and XR formulations were generated at steady-state according to each scenario. The steady state period was defined as days 7, 8 and 9 after treatment initiation. For the adherence scenario (scenario A), doses were administered on a regular basis, as scheduled, up to day 9. For the dose delay, omission, and doubling scenarios (scenarios B–D), dose irregularities were assumed to occur on day 8, with regular dosing being resumed on day 9. For the dose discontinuation scenario (scenario E), treatment was stopped after the last dose on day 8.

### 2.5. Simulation Outcomes

The PK/PD data of each virtual patient was used to calculate the following summary parameters:

(1) *C_max_*: Peak plasma concentration at steady-state;
(2) *C_min_*: Trough plasma concentration at steady-state;
(3) (∂*BPRS)_max_*: Peak %improvement (reduction) in BPRS scores from baseline at steady-state;
(4) (∂*BPRS)_min_*: Trough %improvement (reduction) in BPRS scores from baseline at steady-state;
(5) Δ*C_max_*, Δ*C_min_*, Δ(∂*BPRS)_max_*, Δ(∂*BPRS)_min_*: Δ refers to %change in corresponding parameter value due to irregular administration relative to its value under regular administration conditions. In the case of non-adherence scenarios, the parameters were calculated after the administration of the next scheduled dose;
(6) *T_recovery_*: Time period required to restore regular steady-state plasma levels after the omission of a dose;
(7) *Therapeutic duration*: Time period during which %reduction in BPRS scores from baseline exceeds 14%;
(8) *T_hangover_*: The difference between Therapeutic duration and dosing interval when dose administration is discontinued;
(9) *OR_L_*: Odds ratio of obtaining %reduction in BPRS scores from a baseline of less than 14% in a patient on the XR versus a patient on the IR formulation;
(10) *OR_H_*: Odds ratio of obtaining Quetiapine plasma concentrations of more than 500 µg/L in a patient on the XR versus a patient on the IR formulation.

The statistics on 1000 summary parameters for each case were found. The distributions of parameters calculated in patients on IR and XR formulations were compared using the Kolmogorov–Smirnov test, as implemented in *R* version 3.5.2 (*R* Foundation for Statistical Computing, Vienna, Austria). *p*-value ≤ 0.05 was considered statistically significant. The odds ratios were calculated after the administration of the next scheduled dose in the case of non-adherence scenarios. To calculate the odds ratios, *glm* function in *R* version 3.5.2 (*R* Foundation for Statistical Computing, Vienna, Austria) was used with a binomial distribution error model and logit-link function.

## 3. Results

### 3.1. Simulations of Quetiapine Pre-Steady State PK/PD Profiles and Parameters

**Figure 1:**
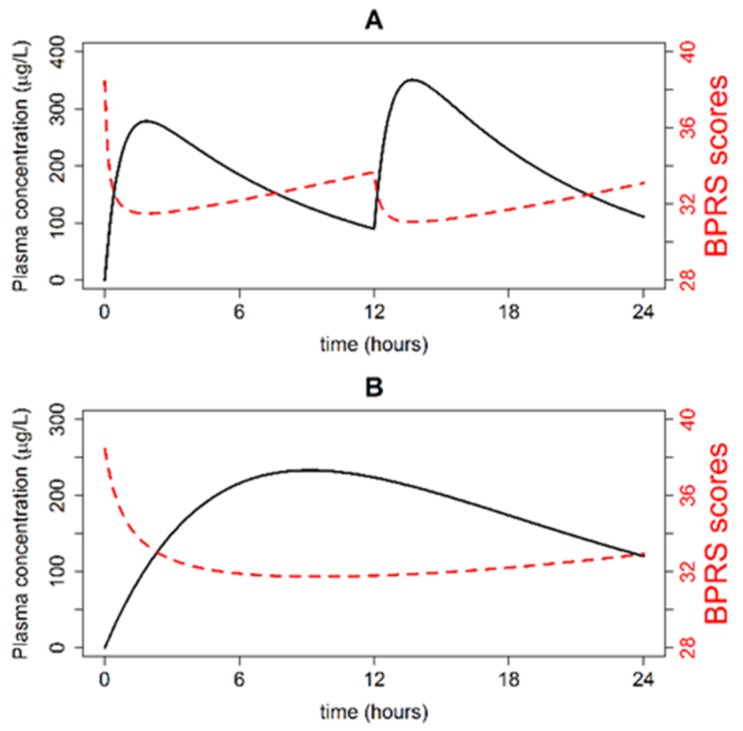
Quetiapine plasma concentration-time (black line) and Brief Psychiatric Rating Scale (BPRS) score time (red line) profiles in a typical patient following the administration of two 200 mg IR doses separated by 12 h (**A**) and one 400 mg XR dose (**B**).

Typical plasma concentration time and BPRS score time profiles were simulated following the oral administration of Seroquel^®^ (two 200 mg doses in a single day) and Seroquel XR^®^ (one 400 mg dose in a single day). The simulations are depicted in Figure 1 (panel A and B). Quetiapine plasma concentration in a typical patient increased to 300 µg/L within 2 h of the IR dose administration, and to 250 µg/L within 8 h of the XR dose administration. BPRS scores in a typical patient ranged between 30 and 38 points for the IR formulation and 32 and 38 points for the XR formulation.

### 3.2. Simulations of Typical Steady-State PK Profiles

**Figure 2:**
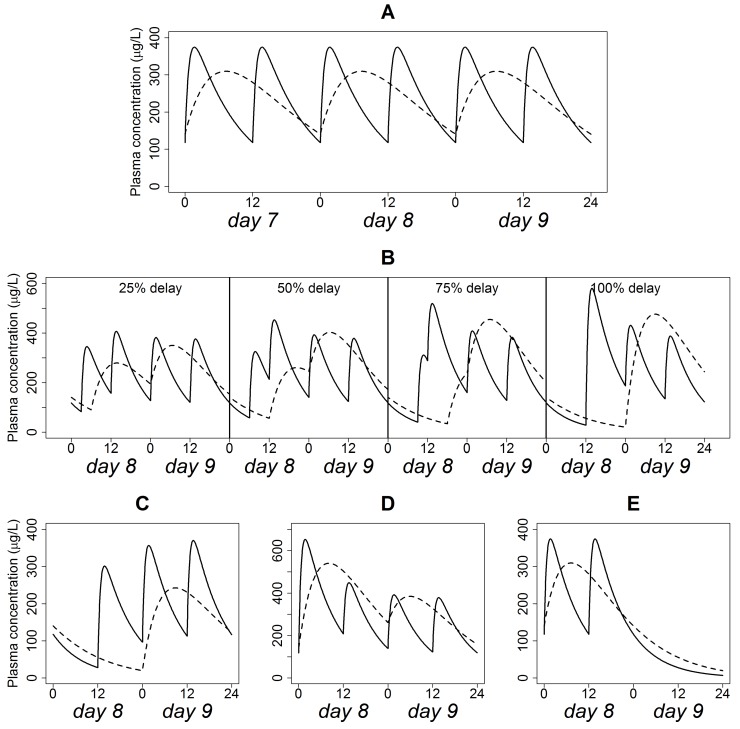
Quetiapine plasma concentration time profiles in a typical patient following the administration of 200 mg BID IR (solid line) and 400 mg QD XR (dashed line) formulations, assuming dose adherence (**A**), delay (**B**), omission (**C**), doubling (**D**) and discontinuation (**E**).

The simulation of Quetiapine plasma concentration time profile at steady state following the administration of the IR formulation (200 mg every 12 h) and XR formulation (400 mg every 24 h), assuming regular dosing is depicted in Figure 2A. Quetiapine plasma levels fell within the range 120–380 µg/L for the IR formulation versus 140–300 µg/L for the XR formulation, suggesting a smaller peak-to-trough fluctuation for the XR formulation.

### 3.3. Simulations of Delayed Doses

The effect of the progressively delayed administration of the IR and XR formulations on the typical plasma concentration time profile is shown in Figure 2B. In these simulations, the interval between two successive doses decreased to 9, 6, 3, and 0 h, respectively, for the IR formulation, and to 18, 12, 6, and 0 h, respectively, for the XR formulation. Before administration of the delayed dose, trough concentrations were below that of steady state. After resuming regular dosing, peak concentrations were higher than that of steady-state. The magnitude of “undershoot” and “overshoot” increased with increased delay interval. However, “undershoot” and “overshoot” in delays up to 50% of the dosing interval did not cause plasma concentrations to fall under 50 µg/L. The 75% delay constituted an inflection point, where trough concentration went below 50 µg/L and peak concentration exceeded 500 µg/L. The effect of “overshoot” was more prominent in the IR formulation than in the XR formulation.

The influence of delayed dosing (relative to regular dosing) on changes in PK and PD parameters following the administration of the IR (200 mg every 12 h) and XR (400 mg every 24 h) formulations is shown in Table 1. The distribution of changes in peak and trough parameters across the range of dose delays was substantially different on the Kolmogorov–Smirnov test (*p* < 0.05).

Compared with adherent dosing, average changes in *C_max_* ranged between 7.9% and 53.4%, with the IR formulation delays, and 7.5% and 45.9% with the XR formulation delays. For *C_min_*, average changes ranged from 1.7% to 12.3% (IR) and 5.2% to 56.5% (XR). The ratio of average change in peak concentration due to delayed administration of XR dose compared to that of IR dose was close to 1, regardless of the delay period. The ratio was approximately 3.2 for the change in trough concentration at the 25%, 50%, and 75% delays, and 4.6 for the 100% delay.

The average change in *(*∂*BPRS)max* (relative to regular dosing) varied between 1.2% and 8.2% with the IR formulation delays and 1.7% and 9.5% with the XR formulation delays. For *(*∂*BPRS)min*, average changes scored 2.6% and 6.5% (IR) and 3.3% and 15% (XR). The ratio of average change in peak %reduction in BPRS scores for the XR to IR formulation was around 1.3 (1.42, 1.31, 1.25, and 1.16 at the 25%, 50%, 75%, and 100% delays, respectively). The ratio ranged between 1.3 and 2.3 for the change in trough% reduction in BPRS scores at the 25% to 100% delays.

### 3.4. Simulation of Omitted Dose

The influence of missing a single dose of the IR (200 mg every 12 h) and XR (400 mg every 24 h) formulations on a typical plasma concentration-time profile is shown in Figure 2C. In this scenario, the interval between the last dose administered prior to non-adherence and the subsequent dose was 12 and 24 h for the IR and XR formulations, respectively. The two-fold difference in the interval is responsible for the quite different “overshoot” in peak concentrations after resumption of scheduled doses. On the other side, the “undershoot” prior to the administration of the scheduled dose produced plasma concentration below 50 µg/L in both formulations.

The changes in *C_max_*, *C_min_*, *(*∂*BPRS)max*, *(*∂*BPRS)min* from regular dosing (when a Ouetiapine dose was completely missed without recovery) are summarized in Table 2. Changes in the trough parameters of the two dosage forms were close, signaling a ratio around 1. However, changes in *(*∂*BPRS)max* and *C_max_* were 2.9- and 3.4-folds, respectively, higher for the XR formulation relative to the IR formulation. The differences between the distributions of the parameters of the two dosage forms were found to be statistically significant (*p* < 0.05).

### 3.5. Simulation of Doubled Dose

This scenario assumes that the dosing interval was strictly followed but one dose is administered twice. The simulated typical plasma concentration-time profiles for the IR (200 mg every 12 h) and XR (400 mg every 24 h) formulations under this scenario are shown in Figure 2D. Concentration “overshoots” in the IR and XR formulations were comparable and allowed the peak plasma concentration to exceed 500 µg/L irrespective of the dosage form type. Associated changes in *C_max_*, *C_min_*, *(*∂*BPRS)max*, *(*∂*BPRS)min* are displayed in Table 2. These changes were 1–3-fold higher for the XR relative to the IR formulation. The difference between the distributions of the parameters of the two dosage forms were found to be statistically significant (*p* < 0.05).

### 3.6. Simulations of Discontinued Administration

Figure 2E shows the typical plasma concentration time patterns following the cessation of the IR (200 mg every 12 h) and XR (400 mg every 24 h) formulation administration. In this scenario, no recovery dose was administered, and even subsequent regular dosing was not resumed. Obviously, the XR formulation exhibited a slower terminal decline rate compared with the IR formulation.

### 3.7. Risk Analysis

**Figure 3:**
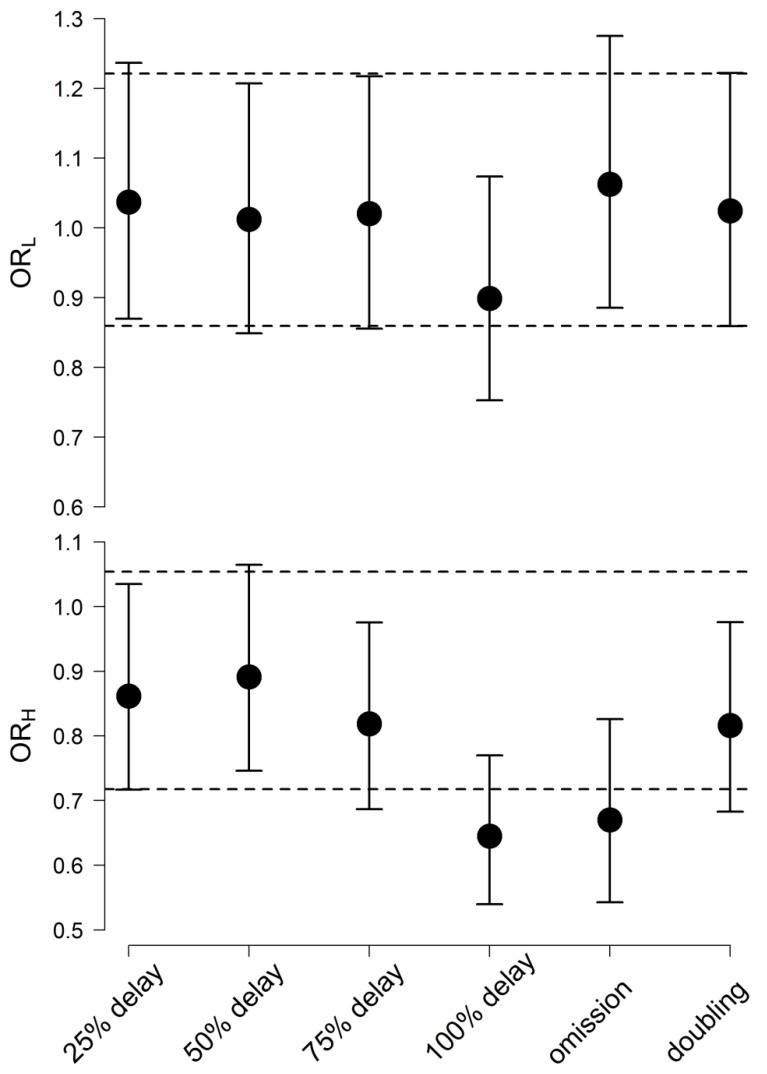
Odds ratio of obtaining %reduction in BPRS scores from baseline less than 14% (upper panel) and Quetiapine plasma concentrations more than 500 µg/L (lower panel) in a patient on the XR versus a patient on the IR formulation at different non-adherence scenarios. The dashed line is the 95% C.I. of the odds ratio, assuming dose adherence.

The results of previous simulations indicated that dose irregularities with the IR (200 mg every 12 h) and XR (400 mg every 24 h) formulations are associated with significantly different effects on Quetiapine plasma concentrations and BPRS scores and Quetiapine key PK and PD parameters as well. To investigate if such differences are clinically relevant, the probability (in terms of odds ratio of event occurrences to non-occurrences) of obtaining a response beyond Quetiapine therapeutic window in adherence and non-adherence scenarios was computed, and the results are depicted in Figure 3.

The average odds ratio of getting %reduction in BPRS scores below 14% ranged between 0.9 and 1.1 when Quetiapine dose was postponed, missed or accidentally doubled. These values fell within the 95% C.I. for the average odds ratio in the case of dose adherence (0.86, 1.22).

When Quetiapine dose was delayed for 25%, 50%, and 75% of the interval, the average odds ratio of plasma concentration exceeding 500 µg/L ranged between 0.8 and 0.9. Close average (0.82) was obtained when two Quetiapine doses were accidentally taken together. These averages were between the 95% Confidence limits when Quetiapine doses were taken as prescribed (0.72,1.1). The average odds ratio in the case of dose delay for 100% of the interval (0.64) and dose omission (0.67) fell below this limit.

### 3.8. Therapeutic Plasma Concentration Hangover

**Figure 4:**
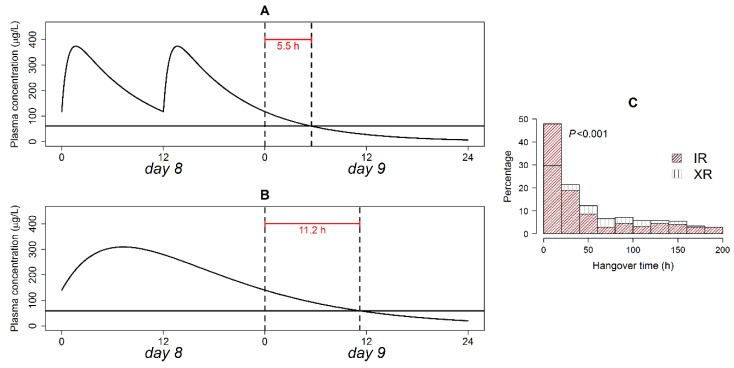
Quetiapine hangover time in a typical patient following the discontinuation of the IR (**A**) and XR (**B**) formulation administration; and relative frequency of hangover times in patients on both formulations (**C**). The solid horizontal line in panels A and B is the plasma concentration associated with a 14% reduction in BPRS score from baseline. *p*-value of the Kolmogorov–Smirnov test is shown in panel (**C**).

The hangover time is how far exactly a drug’s therapeutic effect offset time is from its prescribed dosing interval. The computed hangover time with analysis of its distribution is presented in Figure 4. The XR formulation was associated with a two-fold longer post-interval duration of action compared with the IR formulation in a typical patient (Figure 4, panel A and B). Hangover time exhibited an exponential distribution in the IR and XR formulations (Figure 4C). Even though both formulations had the same type of time distribution (exponential), the distribution rate parameters differed in an extremely significant manner on the Kolmogorov–Smirnov test (*p* < 0.001). Hangover times ranging 0–20 h were more frequent in the IR formulation, while times > 20 h were more frequent in the XR formulations. Plasma therapeutic concentration after treatment cessation was maintained for <24 h in 48% of the patients on the IR, versus only 29.6% of the patients on the XR formulation.

### 3.9. Post-Irregularity Steady-State Recovery

**Figure 5:**
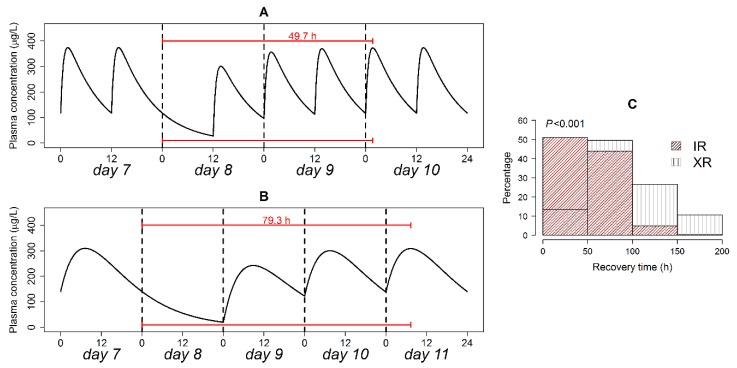
Recovery time in a typical patient following the omission of the IR (**A**) and XR (**B**) formulation doses; and relative frequency of recovery times in patients on both formulations (**C**). *p*-value of the Kolmogorov–Smirnov test is shown in panel (**C**).

The recovery period measures how long exactly it would take the IR (200 mg every 12 h) and XR (400 mg every 24 h) formulations to achieve the exact same steady-state level as achieved prior to dose omission. Recovery time in a typical patient is shown in Figure 5 (panel A and B). For a patient on the XR formulation, recovery time was 1.6-fold longer than for a patient on the IR formulation.

Analysis of the distribution of recovery time is presented in Figure 5C. Recovery time exhibited exponential and quasi-normal distributions for the IR and XR formulations, respectively. The modes of recovery time distributions were at 0–50 and 50–100 h for the IR and XR formulations respectively. On the IR formulation, 51% of the patients restored initial steady state level in <48 h, while only 13.4% achieved this target when the XR formulation was used. Non-parametric comparison of the two distributions using the Kolmogorov–Smirnov test detected a highly significant difference (*p* < 0.001).

## 4. Discussion

The computer modeling and simulation of antiepileptic drugs has been utilized for predicting the consequences of late or missed dosing on the PK of these drugs, and hence for proposing rescue dosing strategies [10,14,23,25,26,27,28]. However, as far as we know, this is the first study to extend this sort of analysis to ASDs. A limitation of the computer modeling and simulation approach is that its validity relies to great extent on the assumptions of the models from which PK and PD responses are generated. Therefore, in this study we were keen to select PK/PD models that involved: (1) a relatively large sample size from diverse population; (2) a wide range of administered doses to ensure acceptable coverage for the drug therapeutic window; (3) a broad sampling time window to warrant complete coverage of the PK and PD profiles; and (4) internal and external validation measures to secure acceptable predictive performance. Based on these criteria, the models by Zhou et al. [12] and Kimko et al. [24] were selected.

The PK profiles of Quetiapine following the administration of the IR and XR formulations were characterized by close trough concentrations. This pertains to the much longer dosing interval relative to the half-life of the drug; the half-life of Quetiapine is approximately 7 h [11,29]. As previously suggested [13], the IR formulation is characterized by a higher peak concentration compared with its XR counterpart. This comes at the expense of greater peak-to-trough fluctuation. The peak concentration is a function of absorption and elimination rate constants, dose and dosing interval. The dose and dosing interval of the XR formulation were twice that of the IR formulation, suggesting that both factors neutralize each other. The elimination rate constant of both formulations was identical, suggesting that the higher peak concentration of the IR formulation is attributed mainly to the absorption rate constant, which was 15 times that of the XR formulation.

Predicted changes in Quetiapine plasma concentration and %reduction in BPRS scores under the influence of non-adherence versus adherence suggest that the IR and XR formulations don’t perform equally when a patient fails to adhere to the prescribed treatment. Delays in the XR formulation dosing had a greater effect on trough plasma concentration and %reduction in BPRS scores than delays with its IR counterpart. Decrements in peak plasma concentration and %reduction in BPRS scores after omitting the XR dose was markedly greater than after omitting the IR dose. The accidental administration of two XR doses produced greater increases in peak and trough plasma concentrations and %reduction in BPRS scores when compared with accidental doublings of IR dose. Accordingly, the IR formulation outperformed its XR counterpart in its swifter effect on some PK and PD parameters in different non-adherence scenarios. However, this does not imply that the clinical utility of the XR formulation is compromised if the patient does not fully adhere to his prescribed regimen. For the most drastic non-adherence events that involve the complete missing or accidental doubling of IR or XR doses, violations of therapeutic window limits in a typical patient were within acceptable values.

The outperformance of the IR formulation is attributed to the simulation conditions adopted in this study. Dosing errors were assumed to happen to 100% and 50% of the total daily dose of Quetiapine XR and IR formulations, respectively. Moreover, the length of the interval between error and recovery events was not the same for both formulations. This finding confirms the sensitivity of simulation results to prior assumptions and conditions. What really matters is that the simulation should mimic what happens in the real world.

In this study, changes in PK and PD metrics were not monitored during the interval of irregularity, but when regular dosing was resumed. Few serious clinical events are expected to surface during the short interval of non-adherence. However, if the PK/PD effects due to non-adherence continue afterwards, undesirable consequences are to be anticipated.

It is noteworthy that peak plasma concentration was the most sensitive parameter to dose irregularities, showing the biggest shifts among PK and PD parameters. Additionally, changes in peak and trough parameters grew with extended delay period, where the largest shift in the parameters was observed when the dose was postponed for 100% of the dosing interval. This can be explained by the degenerating distance between irregularity event time and dose recovery time, leading to higher additive effect on the response.

In our simulations, increasing Quetiapine plasma concentration from 100 to 300 µg/L over a 3 h interval in a typical patient reduced BPRS scores from 34 to 31 points over the same time interval. The non-equal scales of PK and PD responses (3-fold for concentration versus 1.1-fold for BPRS scores) is attributed to the fact that most of the concentrations were higher than the typical Quetiapine *EC50* (83 µg/L), causing BPRS scores to vary near the asymptotic level of the effect–concentration curve. This finding suggests that it is not enough to investigate the effect of dose irregularities on PK alone, even with drugs which don’t require an effect compartment to account for the lag time between systemic availability and therapeutic response. Using PK as a marker for PD is feasible only if the relationship between plasma concentration and PD response is linear (i.e., proportionality stands between concentration and effect).

Analysis of the relative risks associated with dose irregularities revealed that the risk of falling below the lower threshold for therapeutic response in a patient taking XR against a patient taking the IR formulation, assuming dose adherence, did not change much when the patient unintentionally postponed, dropped, or duplicated the dose. The same observation applies to the risk of passing the upper threshold for therapeutic response. The only exception was when Quetiapine dose was delayed for 100% of the interval or completely missed, where the risk was significantly reduced. However, such a reduction is clinically insignificant, since peak plasma concentrations become lower than normal regardless of the dosage form type under these non-adherence scenarios. Based on the outcomes of our clinical trial simulation, it seems that shifting to the XR treatment modality does not aggravate the clinical risks associated with the dose irregularities of the IR modality.

The simulations of the IR and XR formulations demonstrated the dependency of the post-administration duration of effect on the type of dosage form. In orally administered dosage forms, the duration of effect is controlled by the interplay of a drug’s absorption and elimination rates and lower threshold of effective concentration. For IR dosage forms, where peak drug concentration is achieved instantly after administration, the absorption phase is oftentimes ignored, and therapeutic duration is solely determined by elimination half-life. In XR dosage forms, a drug’s release rate is controlled, causing the absorption process to proceed for a longer time compared with IR dosage forms, and hence the peak concentration is achieved later with smaller magnitude. In this case, the elimination rate of the drug is reduced, due to lower plasma concentrations, since first-order elimination is characterized by proportionality of elimination rate to plasma concentration. As a result, the apparent half-life of the XR dosage form is extended, leading to prolonged therapeutic duration.

The extended therapeutic duration is responsible for the prevalence of long hangover times in patients on the XR compared with the IR formulation. The hangover time parameter measures the waiting time until a drug’s plasma concentration level declines to the minimum effective concentration after the next scheduled dose is missed. The parameter reflects the “honeymoon” period, during which the margin of effect continues after missing a dose, and was referred to as a “forgiveness period” elsewhere [22].

To calculate the hangover time, plasma concentration associated with a 14% reduction in BPRS score from baseline was taken as the border line for Quetiapine therapeutic effect. A reduction below14% was associated with a placebo effect in six schizophrenia clinical trials [30]. In this study, the typical plasma concentration associated with 14% reduction in BPRS score was ~70 (µg/L), which is in the reported therapeutic concentration range for Quetiapine (50–500 µg/L) [31].

The typical hangover time was equal to 50% of the dosing interval for both of the IR and XR formulations, suggesting that dose delays with IR and XR formulations up to 6 and 12 h, respectively, are forgivable. However, extending the hangover time beyond 50% of the interval is feasible in the XR formulation through alterations in the drug’s release profile. Customization of the release profile to achieve a desirable post-administration duration of effect is an attribute of a well-designed XR dosage form [22].

The recovery time parameter measures the time interval during which the patient potentially experiences the undesirable effects of missing a scheduled dose. The prevalence of short recovery times in patients on the IR compared with the XR formulation is attributed to a faster absorption rate. The shorter recovery time indicates that the consequences of IR dose missing are quickly managed.

Unlike previously published studies [10,23], this study emphasizes the role of population models and stochastic simulations in comparing the relative performance of IR and XR formulations in non-adherence situations as adopted elsewhere [26,28]. The significantly different distributions of changes in peak and trough plasma concentration and %reduction in BPRS scores found here substantiate the necessity of adopting population simulations rather than individual simulations at typical values. This should be the case even if the drug exhibits linear disposition and broad therapeutic window as Quetiapine.

## 5. Conclusions

Monte-Carlo simulations using validated population PK and PD models predicted that the perturbations in XR and IR dosing are associated with non-equivalent changes in Quetiapine plasma concentrations and BPRS scores, as well as in Quetiapine key PK and PD parameters. However, the simulations predicted that the risks associated with the IR dose irregularities are not worsened when the XR formulation is used instead. Patients on both formulations show robustness to dose delays up to 50% of the prescribed dosing interval. For non-adherence events involving a single dose of either formulation, rescue doses are not necessary. XR formulations surpass IR formulations in their capability of maintaining therapeutic plasma level for longer time periods. IR formulations surpass XR formulations in their capability of restoring regular steady-state plasma levels within shorter time intervals. PK-PD simulations based on models derived from controlled clinical trial data constitute a valuable tool to construct virtual trials that mimic real-world dose non-adherence scenarios to obtain quantitative answers to critical therapeutic inquiries.

## Figures and Tables

**Table 1:** Effect of Quetiapine dose delays on changes in pharmacokinetic (PK) and pharmacodynamic (PD) parameters relative to regular dose administration for the IR and XR formulations. Symbols are defined in text.

Parameter	25% Delay			50% Delay			75% Delay			100% Delay		
	IR	XR	*p* *	IR	XR	*p* *	IR	XR	*p* *	IR	XR	*p* *
Δ*C_max_*	7.9(0.2)	7.5(0.5)	<0.001	19.6(0.2)	18.6(0.6)	<0.001	36.7(0.3)	33.8(0.6)	<0.001	53.4(0.4)	45.9(0.7)	<0.001
Δ*C_min_*	1.7(0.4)	5.2(0.6)	<0.001	4.1(0.4)	12.7(0.7)	<0.001	7.2(0.4)	21(1.1)	<0.001	12.3(0.2)	56.5(0.5)	<0.001
Δ(∂ *BPRS*)*_max_*	1.2(4.6)	1.7(5.1)	<0.001	3.2(4)	4.2(6.2)	<0.001	5.9(3.6)	7.4(7.7)	<0.01	8.2(3.4)	9.5(8.4)	<0.05
Δ(∂ *BPRS*)*_min_*	2.6(1.9)	3.3(2.4)	<0.001	3.9(2.9)	5.3(4)	<0.001	5(3.9)	6.9(7.5)	<0.001	6.5(4.8)	15(8.4)	<0.001

Values are mean (coefficient of variation calculated as standard deviation/mean). * *p*-value of the Kolmogorov–Smirnov test for comparing the distribution of the parameters of the IR and XR formulations.

**Table 2:** Effect of Quetiapine dose omission and doubling on changes in PK and PD parameters relative to regular dose administration for the IR and XR formulations. Symbols are defined in text.

	Dose Omission				Dose Doubling			
	Δ*C_max_*	Δ*C_min_*	Δ(∂ ***BPRS*)*_max_***	Δ(∂ ***BPRS*)*_min_***	Δ*C_max_*	Δ*C_min_*	Δ(∂ ***BPRS*)*_max_***	Δ(∂ ***BPRS*)*_max_***
**IR**	−5.6(0.7)	−74.6(0.2)	−1.6(5.8)	−34.5(5.8)	19.5(0.3)	5.1(0.7)	3(4.2)	4.3(3.3)
**XR**	−19.3(0.5)	−71(0.4)	−4.6(2.7)	−33.3(5)	20.5(0.5)	15.3(0.6)	3.7(3.2)	5.3(6.3)
*p* *	<0.0001	<0.0001	<0.0001	<0.0001	<0.0001	<0.0001	<0.0001	<0.0001

Values are mean (coefficient of variation calculated as standard deviation/mean). * *p*-value of the Kolmogorov–Smirnov test for comparing the distribution of the parameters of the IR and XR formulations.

## Supplementary Materials

The following are available online at https://www.mdpi.com/1999-4923/12/4/297/s1, *R*-codes for stochastic simulation of Quetiapine plasma concentrations, BPRS scores, and %Reduction in BPRS scores from baseline following administration of IR and XR formulations assuming dose adherence, delay, omission, and doubling.
