# Supplementary material for: Changing the Drug Delivery System: Does it Add to Non-Compliance Ramifications Control? A Simulation Study on the Pharmacokinetics and Pharmacodynamics of Atypical Antipsychotic Drug
Source: Pharmaceutics. 2020 Mar 25;12(4):297. doi: 10.3390/pharmaceutics12040297 (PMC7238021; doi:10.3390/pharmaceutics12040297)
Supplement: Supplementary file 1 [file pharmaceutics-12-00297-s001.pdf]

pharmaceutics

Supplementary Materials: Changing the Drug Delivery System: Does it Add to Non-Compliance Ramifications Control? A Simulation Study on the Pharmacokinetics and Pharmacodynamics of Atypical Antipsychotic Drug

Mohammed H. Elkomy

R-codes for stochastic simulation of Quetiapine plasma concentrations, BPRS scores, and %Reduction in BPRS scores from baseline following administration of IR and XR formulations assuming dose adherence, delay, omission, and doubling

```
#########################################################
##################INNER FUNCTIONS###########################
##############################################################
model<-function(t,dose,par,form){
Ke<-par[1]
Ka_ir<-par[2]
Ka_xr<-par[3]
V<-par[4]
Ka<-Ka_ir
if(form==1)Ka<-Ka_xr

A<-dose*Ka/(V*(Ka-Ke))
C<-A*(exp(-Ke*t)-exp(-Ka*t))
return(C)
}
###########################
one.day.resp<-function(time,day.dose,day.tau,par,form){
day.tau<-day.tau[!is.na(day.dose)]
day.dose<-day.dose[!is.na(day.dose)]

Cout<-0

for(i in 1:length(day.tau)){
tscale<-time-day.tau[i]
conc<-model(tscale,day.dose[i],par,form)
conc[time<day.tau[i]]<-0
Cout<-Cout+conc
}
return(Cout)
}
#####################################
###########################################################
###############OUTER FUNCTION##############################
```

```
#########################################################
#tsld: Vector of time since last dose
#dose.by.day.tau: Matrix of administered doses by day and tau.
#tau is the vector of administration times per day assuming that drug administration has
started on that day
#start.record: The first day to record concentration at.
#stop.record: The last day to record concentration at.
#par: Vector of PK parameters (Ke, Ka_ir, Ka_xr, V)
#form: 0 if IR formulation and 1 if XR formulation
tot.resp<-function(tsld,dose.by.day.tau,start.record,stop.record,par,form){
day.tau<-as.numeric(as.vector(colnames(dose.by.day.tau)))
Cout<-NULL
tout<-NULL

for(j in start.record:stop.record){
cum<-0
for(i in j:1){
time<-((i-1)*24)+tsld
day.dose<-as.vector(dose.by.day.tau[j-i+1,])
resp<-one.day.resp(time,day.dose,day.tau,par,form)
cum<-cum+resp
}
Cout<-cbind(Cout,cum)
tout<-cbind(tout,((j-1)*24)+tsld)
}
fin<-data.frame(day=rep(start.record:stop.record,each=length(tsld)),
tsld=rep(tsld,stop.record-start.record+1),
time=as.vector(tout),conc=as.vector(Cout))
#fin<-fin[!duplicated(fin$time),]
BPRS<-par[5]+par[8]*fin$time-((par[6]*fin$conc)/(par[7]+fin$conc))
BPRSred<-((par[5]-BPRS)/par[5])*100
fin<-data.frame(fin,BPRS=BPRS,BPRSred=BPRSred)
fin<-data.frame(Form=rep(form,nrow(fin)),fin)
return(fin)
}
#########################################################
############PARAMETER GENERATION FUNCTION####################
par.gen<-function(n){
pk.avg<-c(0.12,1.46,0.1,573.7)
pk.sd<-c(0.4,0.75,1.5,0.47)
pd.avg<-c(3.65,2.2,4.42,-0.008)
pd.var<-c(0.27,0.41,1.77,0.0001)
par.mat<-c(pk.avg,exp(pd.avg[1:3]),pd.avg[4])
if(n>1){
par.mat<-NULL
for(i in 1:n){
pk.par<-exp(rnorm(rep(1,4),log(pk.avg),pk.sd)))
pd.par<-exp(rnorm(rep(1,4),pd.avg,sqrt(pd.var)))
```

```
pd.par[4]<-log(pd.par[4])
par<-c(pk.par,pd.par)
par.mat<-rbind(par.mat,par)
}
}
return(par.mat)
}
##########END FUNCTIONS######################################
##########################################################
par.list<-par.gen(1000)
out.fin<-NULL
for(i in 1:nrow(par.list)){
print(i)
par<-par.list[i,]
######################################################################
#########Scenario A: Dose Adherence##################################
#Simulate response to IR Formulation at days 7-9.
#Similar dose regimen (200 mg q 12 h) is administered till day 9
###############################################
doses<-rep(c(200000,200000),9)
tau<-rep(c(0,12),9)
days<-rep(1:9,each=2)

dose.by.day.tau<-tapply(doses,list(days,tau),unique)

tsld<-seq(0,24,by=0.1)
out.IR<-tot.resp(tsld,dose.by.day.tau,9,9,par,0)
out.IR<-data.frame(Scenario=1,ID=i,out.IR)
##############################################
#Simulate response to XR Formulation at days 7-9.
#Similar dose regimen (400 mg q 24 h) is administered till day 9
###############################################
doses<-rep(400000,9)
tau<-rep(0,9)
days<-1:9

dose.by.day.tau<-tapply(doses,list(days,tau),unique)

tsld<-seq(0,24,by=0.1)
out.XR<-tot.resp(tsld,dose.by.day.tau,9,9,par,1)
out.XR<-data.frame(Scenario=1,ID=i,out.XR)
##############################################
out1<-rbind(out.IR,out.XR)
#############################################
#############################################
############################################################
#########Scenario B: Dose Delay for 25% of the
interval##################################
```

```
#Simulate response to IR Formulation at days 7-9.
#Similar dose regimen (200 mg q 12 h) is administered till day 7
#then a different dose regimen is administered on day 8 (200 mg after 0 and 15 hours)
#then the regular dosing is continued on day 9 (200 mg q 12 h)
##############################################
regular.dose1<-rep(c(200000,200000),7)
odd.dose<-c(200000,200000)
regular.dose2<-c(200000,200000)
doses<-c(regular.dose1,odd.dose,regular.dose2)

regular.tau1<-rep(c(0,12),7)
odd.tau<-c(0,15)
regular.tau2<-c(0,12)
tau<-c(regular.tau1,odd.tau,regular.tau2)

regular.days1<-rep(1:7,each=2)
odd.days<-rep(8,2)
regular.days2<-rep(9,2)
days<-c(regular.days1,odd.days,regular.days2)

dose.by.day.tau<-tapply(doses,list(days,tau),unique)

tsld<-seq(0,24,by=0.1)
out.IR<-tot.resp(tsld,dose.by.day.tau,9,9,par,0)
out.IR<-data.frame(Scenario=2.1,ID=i,out.IR)
#############################################
#Simulate response to XR Formulation at days 7-9.
#Similar dose regimen (400 mg q 24 h) is administered till day 7
#then a different dose regimen is administered on day 8 (400 mg after 6 hours)
#then the regular dosing is continued on day 9 (400 mg at 0 hours)
##############################################
regular.dose1<-rep(400000,7)
odd.dose<-400000
regular.dose2<-400000
doses<-c(regular.dose1,odd.dose,regular.dose2)

regular.tau1<-rep(0,7)
odd.tau<-6
regular.tau2<-0
tau<-c(regular.tau1,odd.tau,regular.tau2)

regular.days1<-1:7
odd.days<-8
regular.days2<-9
days<-c(regular.days1,odd.days,regular.days2)

dose.by.day.tau<-tapply(doses,list(days,tau),unique)
```

```
tsld<-seq(0,24,by=0.1)
out.XR<-tot.resp(tsld,dose.by.day.tau,9,9,par,1)
out.XR<-data.frame(Scenario=2.1,ID=i,out.XR)
#############################################
out2.1<-rbind(out.IR,out.XR)
############################################
##########################################################
##########################################################
#########Scenario C: Dose Omission##################################
#Simulate response to IR Formulation at days 7-9.
#Similar dose regimen (200 mg q 12 h) is administered till day 7
#then a different dose regimen is administered on day 8 (200 mg after 0 hours only)
#then the regular dosing is continued on day 9 (200 mg q 12 h)
##############################################
regular.dose1<-rep(c(200000,200000),7)
odd.dose<-200000
regular.dose2<-c(200000,200000)
doses<-c(regular.dose1,odd.dose,regular.dose2)

regular.tau1<-rep(c(0,12),7)
odd.tau<-0
regular.tau2<-c(0,12)
tau<-c(regular.tau1,odd.tau,regular.tau2)

regular.days1<-rep(1:7,each=2)
odd.days<-8
regular.days2<-rep(9,2)
days<-c(regular.days1,odd.days,regular.days2)

dose.by.day.tau<-tapply(doses,list(days,tau),unique)

tsld<-seq(0,24,by=0.1)
out.IR<-tot.resp(tsld,dose.by.day.tau,9,9,par,0)
out.IR<-data.frame(Scenario=3,ID=i,out.IR)
#############################################
#Simulate response to XR Formulation at days 7-9.
#Similar dose regimen (400 mg q 24 h) is administered till day 7
#then a different dose regimen is administered on day 8 (No dose is administered at all)
#then the regular dosing is continued on day 9 (400 mg at 0 hours)
##############################################
regular.dose1<-rep(400000,7)
odd.dose<-0
regular.dose2<-400000
doses<-c(regular.dose1,odd.dose,regular.dose2)

regular.tau1<-rep(0,7)
odd.tau<-0
regular.tau2<-0
```

```
tau<-c(regular.tau1,odd.tau,regular.tau2)

regular.days1<-1:7
odd.days<-8
regular.days2<-9
days<-c(regular.days1,odd.days,regular.days2)

dose.by.day.tau<-tapply(doses,list(days,tau),unique)

tsld<-seq(0,24,by=0.1)
out.XR<-tot.resp(tsld,dose.by.day.tau,9,9,par,1)
out.XR<-data.frame(Scenario=3,ID=i,out.XR)
###############################################
out3<-rbind(out.IR,out.XR)
##############################################
#############################################################
#########Scenario D: Dose Doubling###################################
#Simulate response to IR Formulation at days 7-9.
#Similar dose regimen (200 mg q 12 h) is administered till day 7
#then a different dose regimen is administered on day 8 (200 mg after 0 hour and 400 mg after 12 hours)
#then the regular dosing is continued on day 9 (200 mg q 12 h)
################################################
regular.dose1<-rep(c(200000,200000),7)
odd.dose<-c(200000,400000)
regular.dose2<-c(200000,200000)
doses<-c(regular.dose1,odd.dose,regular.dose2)

regular.tau1<-rep(c(0,12),7)
odd.tau<-c(0,12)
regular.tau2<-c(0,12)
tau<-c(regular.tau1,odd.tau,regular.tau2)

regular.days1<-rep(1:7,each=2)
odd.days<-rep(8,2)
regular.days2<-rep(9,2)
days<-c(regular.days1,odd.days,regular.days2)

dose.by.day.tau<-tapply(doses,list(days,tau),unique)

tsld<-seq(0,24,by=0.1)
out.IR<-tot.resp(tsld,dose.by.day.tau,9,9,par,0)
out.IR<-data.frame(Scenario=4,ID=i,out.IR)
###############################################
#Simulate response to XR Formulation at days 7-9.
#Similar dose regimen (400 mg q 24 h) is administered till day 7
#then a different dose regimen is administered on day 8 (800 mg at 0 hours)
#then the regular dosing is continued on day 9 (400 mg at 0 hours)
```

```
##############################################
regular.dose1<-rep(400000,7)
odd.dose<-800000
regular.dose2<-400000
doses<-c(regular.dose1,odd.dose,regular.dose2)

regular.tau1<-rep(0,7)
odd.tau<-0
regular.tau2<-0
tau<-c(regular.tau1,odd.tau,regular.tau2)

regular.days1<-1:7
odd.days<-8
regular.days2<-9
days<-c(regular.days1,odd.days,regular.days2)

dose.by.day.tau<-tapply(doses,list(days,tau),unique)

tsld<-seq(0,24,by=0.1)
out.XR<-tot.resp(tsld,dose.by.day.tau,9,9,par,1)
out.XR<-data.frame(Scenario=4,ID=i,out.XR)
#############################################
out4<-rbind(out.IR,out.XR)
############################################
###########################################################
################################################
out<-rbind(out1,out2.1,out3,out4)
out.fin<-rbind(out.fin,out)
}
####################################################################
write.csv (out.fin,file="D://---/analysis/SimData.csv",row.names=FALSE)
#####################END OF CODE################################
```
